# HIV Prophylaxis in High Risk Newborns: An Examination of Sociodemographic Factors in an Inner City Context

**DOI:** 10.1155/2016/2782786

**Published:** 2016-04-03

**Authors:** Zenita Alidina, Anne E. Wormsbecker, Marcelo Urquia, Jay MacGillivray, Evan Taerk, Mark H. Yudin, Douglas M. Campbell

**Affiliations:** ^1^St. Michael's Hospital, University of Toronto, Toronto, ON, Canada M5B 1W8; ^2^Department of Pediatrics, Hospital for Sick Children, Toronto, ON, Canada M5G 1X8; ^3^Department of Medicine, University of Toronto, Toronto, ON, Canada M5S 1A8; ^4^Dalla Lana School of Public Health, University of Toronto, Toronto, ON, Canada M5T 3M7; ^5^Keenan Research Centre of the Li Ka Shing Knowledge Institute, St. Michael's Hospital, Toronto, ON, Canada M5B 1T8

## Abstract

*Background.* Perinatal HIV transmission is less than 1% with antiretroviral (ARV) prophylaxis. Transmission risk appears higher in “high risk” dyads, yet this is not well defined, possibly exposing more infants to combination ARV compared with standard care.* Objective.* To describe characteristics of mother-infant dyads where infants received ARVs and how these characteristics relate to specific ARV regimens.* Methods.* Retrospective chart review of ARV-receiving newborns at St. Michael's Hospital from 2007 to 2012 (and their mothers). Numerical and categorical variables were analyzed using *t*-tests/ANOVA *F*-tests and Fisher's exact tests, respectively.* Results.* Maternal HIV status at delivery was as follows: 69% positive and 24% unknown. Maternal factors significantly associated with newborn-triple therapy are Canadian origin, substance abuse, unstable housing, lost custody of previous children, and sex work. Neonatal factors are child protective services involvement, NICU, and lengthier admission. Maternal factors associated with monotherapy are African origin, HIV-positive, employment, and education. Further analysis based on maternal presentation at delivery demonstrated unequal distribution of many aforementioned factors.* Discussion.* This cohort revealed associations between particular factors and newborn-monotherapy or triple therapy that exist, suggesting that sociodemographic factors may influence the choice of ARV regimen. Canadian perinatal HIV transmission guidelines should qualify how to risk stratify newborns and consider use of rapid HIV antibody testing.

## 1. Introduction

The risk of perinatal transmission can be reduced to as low as 0.4% in developed countries, with access to antiretroviral (ARV) treatment for both mothers and newborns. However, due to HIV drug resistance, high viral loads, and unrecognized HIV infection late in pregnancy, cases of HIV-infected infants continue to be reported [1, 2]. Between 1984 and 2013, the largest proportion of cases of perinatal HIV exposure in Canada occurred in Ontario, and as of 2011, 62.5% of these Ontarian mothers originated from HIV endemic countries [3, 4]. In 2013, the Canadian Perinatal HIV Surveillance Program recorded 201 cases of perinatal HIV exposure (infants born to HIV-positive women), with 2 confirmed cases of HIV-positive infants and 22 that remain unconfirmed [3].

The primary treatment strategy for perinatally exposed infants has been zidovudine (AZT) monotherapy for almost 20 years [5]. Additional ARVs are used in prophylactic treatment of newborns, largely prescribed based on the perceived “risk” of perinatal transmission. Patient characteristics that often infer “high risk” of transmission include high viral load at delivery or late in pregnancy; country of origin (i.e., if endemic with HIV); intravenous drug use (IDU); poor maternal ARV compliance; preterm delivery; late presentation in pregnancy or no prenatal care; coinfections, such as chlamydia; unprotected sex with multiple partners; and unprotected sexual contact with known HIV-infected partner(s) [1, 2, 6–10]. Although the literature identifies these factors as key variables, there is no clearly defined stratification of risk. The lack of defining criteria to identify high risk patients can lead to a subjective determination of which newborns warrant mono-, dual, or triple therapy.

Recommendations from the US Department of Health and Human Services endorse that infants at high risk of HIV exposure receive dual therapy with AZT and nevirapine (NVP) [11]. Ontario recommendations support the use of triple ARV therapy with AZT, lamivudine (3TC), and NVP as the preferred treatment for newborns of a high risk dyad [12–14]. Triple therapy may be associated with increased side effects in newborns when compared directly to dual therapy, such as anemia and neutropenia [6], and rarely results in lactic acidosis, mitochondrial dysfunction, or altered lymphocyte development [7, 15–17]. The increased burden of care and costs placed on caregivers and parents that results when adding multiple ARVs to a newborn's treatment regimen must also be considered given the challenge of compliance and administrating additional medication.

Through this study, we sought to determine if newborns who receive multiple ARVs, and their mothers, are more likely to have specific characteristics that could contribute to a heightened perceived risk level compared to newborns who receive ARV monotherapy and their mothers. Our primary objectives were (1) to describe the characteristics of mother-infant dyads, for which the infant is treated with ARV therapy, and (2) to explore maternal and newborn characteristics, including sociodemographic factors, related to specific ARV regimens and specific mother-infant dyads.

## 2. Methods

### 2.1. Study Population and Data Collection

St. Michael's Hospital (SMH) is a large, Canadian, inner city, tertiary hospital that provides care for the majority of perinatal cases of HIV in the Greater Toronto Area in Ontario. Maternal care for these cases is facilitated by the Positive Pregnancy Programme (P3), which is led by an interprofessional obstetrics and midwifery team to offer support and integrated care to HIV-positive pregnant women. Follow-up care for newborns on ARVs is then provided by the infectious disease team at the Hospital for Sick Children (HSC) on an outpatient basis. We used the neonatal HIV databases at SMH and HSC to identify all newborns delivered at SMH between January 1, 2007, and August 31, 2012, who received ARVs at birth. A retrospective electronic chart review completed the charts of both newborns and their mothers identified as eligible mother-infant dyads. The study was approved by the Research Ethics Board at St. Michael's Hospital.

### 2.2. Study Outcomes

Data collection included the following newborn characteristics: gestational age at birth, ARVs received (newborn-monotherapy versus newborn-triple therapy); rationale for triple therapy; toxicology results; NICU admission rates; length of stay in hospital; and identifying the involvement of child protective services (CPS). Maternal characteristics reviewed included age; country of origin; number of prenatal visits; receipt of intrapartum AZT; partner involvement; substance use; smoking status; stability of housing; Ontario Health Insurance Plan (OHIP) coverage; social work involvement; CPS involvement with previous children; sex work; employment status; and education level.

### 2.3. Secondary Analysis

A secondary analysis was performed to look at the spread of study outcomes in four distinct groups of mother-infant dyads. These groups were identified based on clinical features of the mother in the dyad as they would most often present to the clinician in the labour and delivery room deciding on neonatal treatment. Group 1 consisted of mothers who were known to be HIV-positive at delivery, stable on ARV therapy with an undetectable viral loads (<50 copies/mL) reflective of excellent prenatal care. Group 2 had mothers who were known to be HIV-positive at delivery; however, they were not stable on ARV therapy and/or had a detectable viral load at delivery. Group 3 contained mothers who presented to their delivery without a prior HIV test result, and Group 4 were mothers with a previous HIV-negative but were felt to be at “high risk.” For the purpose of the secondary analysis “high risk” was defined as a mother who had three or more of the follow characteristics: age of 20 years or younger, two or less prenatal visits, no partner involvement, known or suspected substance use, unstable housing (living in a shelter or being homeless), no OHP coverage, social work involvement, CPS involvement with previous children, sex work, unemployed, and not having completed high school.

In creating the groups for the secondary analysis dyads were excluded if the reason for newborn HIV prophylaxis was driven by the mother's partner being HIV-positive while the mother herself was HIV-negative, and an additional dyad was excluded as there was no ascertainable viral load data for the duration of the mother's pregnancy at the time the retrospective chart review was performed. This consisted of a total of 4 excluded dyads and the purpose was such that they would not fit within the four groups outlined above.

### 2.4. Statistical Methods

Descriptive statistics were calculated for the eligible mother-infants dyads and for newborn ARV regimen subgroups. Categorical variables and continuous maternal characteristics were compared between the two ARV regimen groups using Fisher's exact tests and two-sided *t*-tests, respectively. For the secondary analysis of the four groups as defined by different maternal presentations at delivery, categorical variables were analyzed using Fischer's exact tests and continuous variables were analyzed using ANOVA *F*-tests. Results were reported as statistically significant at *p* < 0.05. If subjects had missing data in a single category, these were excluded for the purpose of statistical analysis when performing Fisher's exact tests. Data was managed and stored in Microsoft Excel 2010 (Version 14.0.7106.5003) and SAS® (Version 9.3, Cary, NC) software was used for analysis.

## 3. Results

### 3.1. Patient Characteristics

A total of 122 eligible mother-infant dyads were identified. Of these 122 pairs, 84 (69%) had known HIV-positive mothers, 9 (7%) had known HIV-negative mothers, and 29 (24%) were of unknown status. In the entire cohort, almost 50% were of African origin (*n* = 60). Many women had high risk sociodemographic characteristics: 34% were engaged in substance abuse (*n* = 42); 20% were homeless or living in a shelter (*n* = 25); 20% did not have health insurance through the Ontario Health Insurance Plan (OHIP) (*n* = 25); 41% did not have a partner involved (*n* = 50); 20% had lost custody of their previous children (*n* = 25); 12% were known to be engaging in sex work (*n* = 15); and 57% were unemployed (*n* = 70) (Table 1). Of the 86 women who were identified as HIV positive, 72% had undetectable viral loads (*n* = 62), and the majority had received routine AZT prophylaxis during labour and delivery (95%, *n* = 82).

Analysis of the data collected for newborns showed the following characteristics: 30% (*n* = 37) received triple therapy; 32% (*n* = 39) were admitted to the NICU, of which 49% were preterm (less than 37 weeks' gestation, *n* = 19); 34% (*n* = 41) had child protective services involved; and 27% (*n* = 33) had positive toxicology screens (cocaine was reported in 31/33 cases). The three most common reasons for the use of triple therapy, in order of declining frequency, were maternal high risk behaviour, having no specific rationale provided, and high maternal viral load (Table 2).

### 3.2. Characteristics Related to Newborn ARV Regimens

**Figure 1:**
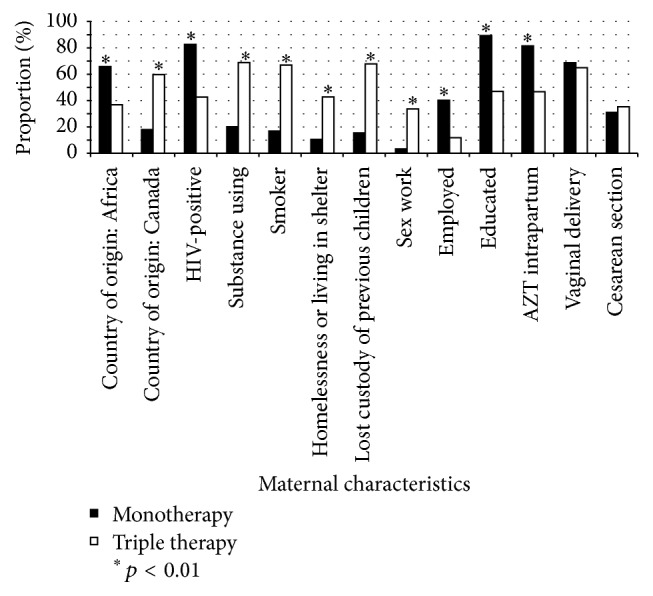
Proportion with selected maternal characteristics in newborn-monotherapy versus newborn-triple therapy dyads.

Comparisons of differences in maternal characteristics between dyads in which newborns received triple therapy and dyads in which newborns received monotherapy are shown in Table 3 and Figure 1. Maternal factors that were significantly associated with triple therapy (*p* < 0.05) included younger age; Canadian country of origin; substance use; positive smoking status; homelessness or living in a shelter; social work referral in hospital; loss of custody of previous children; and sex work (Table 3 and Figure 1). Neonatal factors significantly associated with triple therapy (*p* < 0.05) were positive toxicology screen, NICU admission, increased length of stay in hospital, and child protective services involvement and apprehension (Table 4).

Maternal factors significantly (*p* < 0.01) associated with newborn-monotherapy included African country of origin; known HIV-positive status; viral load of less than 50 copies/mL; increased number of prenatal visits; residence in their own home; employment; at least a grade 12 education; and receipt of intrapartum AZT (Table 3 and Figure 1). The only neonatal factor significantly associated with monotherapy (*p* < 0.01) was increased gestational age (Table 4).

### 3.3. Secondary Analysis by Different Maternal Presentations at Delivery

This analysis examined dyads based on maternal presentation at delivery which was divided into four groups. Group 1 consisted of mothers who were known to be HIV-positive at delivery, stable on ARV therapy with an undetectable viral loads (<50 copies/mL) reflective of excellent prenatal care. Group 2 had mothers who were known to be HIV-positive at delivery; however, they were not stable on ARV therapy and/or had a detectable viral load at delivery. Group 3 contained mothers who presented to their delivery without a prior HIV test result, and Group 4 were mothers with a previous HIV-negative but were felt to be at “high risk” (defined above).

A total of 118 dyads were included in the secondary analysis. 52% of dyads fell into Group 1 (*n* = 62), 19% in Group 2 (*n* = 22), 17% in Group 3 (*n* = 20), and 12% in Group 4 (*n* = 14). Within these groups the distribution of newborns that received triple ARV therapy was significantly different (*p* < 0.01) with the highest proportion of newborns receiving triple therapy in Group 4 at 71% and the lowest in Group 1 at 3% (Table 5).

Statistical differences in the distribution of maternal and neonatal characteristics amongst the four groups appear to be most attributable to the distributions in Groups 1 and 4. Statistically significant associations that may be attributable to Group 1 based on the proportion for that test being particularly high or low included the following: on average they were older in age, 97% lived in their own home, 97% had custody of their previous children, none were involved in sex work (Table 6), their newborns tended to have the highest gestational age and birth weight (although no association was found with regard to SGA), and the newborns spent the least amount of time in hospital with a mean length of stay of three days (Table 5). Group 2 was found to have the highest proportion of mothers with at least a grade 12 education (100%), followed closely by Group 1 mothers at 90%. Group 3 may have driven the statistical significance behind the association with maternal age where, in contrast to Group 1, mothers were youngest in Group 3, and 100% of these dyads had social work involvement at the time of delivery. Group 4 may also have been responsible for this association as this group also had 100% social work involvement. Further characteristics that were found to be statistically significant in the secondary analysis, potentially attributable to the proportions in Group 4, were as follows: 100% of the mothers were from Canada, they had the least number of prenatal visits, 100% were found to be HIV-negative postdelivery, 75% of mothers presented to their delivery without the involvement of the partner, and 100% were known or suspected of using substance, as well as smoking, being unemployed, and not receiving AZT intrapartum (Table 6). With respect to the spread of neonatal characteristics in Group 4 that may have accounted for statistically significant associations, these newborns had the lowest gestational age and birth weight, they were more likely to be admitted to the NICU (86%), and 100% of the newborns had CPS involvement (Table 5).

In examining the characteristics that were found to be significantly associated in our initial analysis of newborns receiving triple therapy as compared to those that received monotherapy, many of the similar characteristics tended to be unequally distributed amongst the four groups in our secondary analysis. All of the factors found to be significant in the initial analysis are listed above with the exception of positive newborn toxicology screen which was not significant in the secondary analysis (Table 5).

## 4. Discussion

In this study maternal factors associated with newborn-triple therapy were related to social determinants of health including substance use, residence (homelessness or living in a shelter), loss of custody of previous children, and a history of sex trade work. Choice of monotherapy treatment in the newborn was associated with reassuring maternal socioeconomic characteristics such as enrolment in an ongoing prenatal program, living in their own home, employment, at least a grade 12 education, and receipt of intrapartum AZT. Patients followed up closely in a specialized prenatal program rarely required triple therapy. When examining these same factors based on the maternal clinical presentation at delivery there continues to be an uneven distribution of these characteristics that were found to be different. Mothers known to be HIV-positive and stable on ARVs with an undetectable viral load and excellent prenatal care had newborn treatment plans which were similar to those dyads whose newborns received monotherapy. Our data as a whole suggests that social determinants of health may influence choice of ARV regimen in newborn HIV prophylaxis.

Interestingly, in this study, newborns that received triple therapy had significantly higher rates of NICU admission and increased length of stay in hospital and are more likely to have child protective services involvement and subsequent apprehension. These newborns characteristics could be related to the presence of maternal social risk factors, as evidenced by the same factors being significantly associated and presumably driven by the mothers who met criteria for Group 4 in the secondary analysis. NICU admission and increased length of stay are unlikely related to specific use of triple therapy and may have stemmed more from the high risk socioeconomic nature of these mothers resulting in premature delivery, low birth weight, the need for management of substance withdrawal, and other comorbid conditions.

Specific social risk factors, such as sex work and drug use, are associated with a woman's HIV risk status. If these particular factors led to HIV acquisition during pregnancy they would indeed be linked to increased risk of perinatal HIV transmission, necessitating the use of triple therapy for newborns [1, 2, 7–9]. Further probing on admission histories is needed to determine if drug exposure was via IDU, and if barrier protection was used during intercourse. Presence of these factors elevates the risk of the mother acquiring HIV and, without receipt of appropriate treatment, necessitates the use of a multi-ARV regimen for the exposed newborn.

Our study linking newborn prophylaxis regimens for HIV-exposed newborns to maternal social determinants of health has its limitations. Data collection was retrospective and relied on documentation by clinicians on call, which may not have indicated an explicit reason as to why triple therapy was chosen. Clinical information used to contribute to newborn treatment decisions may not have been documented or available at the time of the decision (e.g., high risk sexual profile of mothers). This could be due to a lack of documentation or a lack of thorough history-taking by either the obstetric and/or pediatric clinician involved. Secondly, social work notes, which often are more complete in documenting social determinants of health, are not consistently available or sometimes not accessible via the electronic hospital chart. Our study does not attempt to address effectiveness or side effects of monotherapy versus triple therapy and we did not follow the newborns to determine their ultimate HIV status. Although outcomes postdelivery were not a focus of our study, these may provide further valuable insight as to the implications of different ARV regimens and the decision-making process. Finally, generalizability is limited given that this is a single centre study in urban, inner city Toronto and in performing our secondary analysis the division of a single centre study into four groups presented a challenge in being able to have the necessary power to compare factors between the groups for statistical significance.

Despite limitations, our results do suggest that maternal social factors seem to play a role in influencing risk assessment and choice of ARV for newborns. In light of the lack of detailed formal Canadian clinical practice guidelines regarding the treatment of perinatally HIV-exposed newborns, physicians may rely on US recommendations and/or recommendations formulated in Ontario. These provide conflicting information on the use of dual versus triple therapy and it remains unclear as to what constitutes high risk [11–14]. Given that we found different social determinants of health to be associated with the two ARV regimens for newborns, further investigation into exactly which of these characteristics ultimately influence treatment decisions and/or are associated with increased risk of HIV transmission will help shape future practice guidelines. In particular, assessment of how sociodemographic risk factors influence newborn treatment decisions will help guide clinicians when faced with a mother who is HIV-negative or whose status is unknown, but who is at risk of acquiring HIV during her pregnancy. Individual practitioners may interpret similar sociodemographic data differently; for some a newborn may be at “high risk,” and for others the same information would constitute being at “low risk.”

Changing practice guidelines based on risk status of dyads is already being undertaken at other sites. In the United Kingdom and Ireland there has been a movement towards reducing AZT monotherapy regimens from 6 weeks to 4 weeks if dyads are deemed low risk. These European guidelines define high risk for perinatal transmission based on medical factors such as premature labour, absence of peripartum prophylaxis, and detectable viral load at delivery. The more conservative approach to monotherapy does not appear to negatively impact perinatal transmission rates and in fact serves to reduce medication burden and potential side effects to newborns [18–20]. The emphasis is on ascertaining risk status based on medical factors as opposed to socioeconomic factors. This strategy may be something to incorporate in the North American, and specifically Canadian, context as well.

To allow physicians to assess perinatal HIV transmission risk much more accurately than by way of considering sociodemographic risk factors, routine rapid HIV antibody testing for pregnant women of unknown HIV status in labour could also provide more information for practitioners. This practice is recommended by several regulatory bodies including the American Academy of Pediatrics, the Centre for Disease Control, the US Department of Health and Human Services, the German-Austrian Recommendations for HIV Therapy in Pregnancy, and Ontario recommendations to expedite treatment decision-making regarding intrapartum ARVs and newborn prophylaxis [7, 11, 13, 14, 21, 22].

Concerns around feasibility and effects on time to receipt of ARV prophylaxis are important considerations. Pilot studies demonstrate widespread implementation of rapid HIV antibody testing in pregnant women that present to labour and delivery is possible [23] and there appear to be no significant differences in perinatal HIV transmission rate if AZT is started within 48 hours of life as compared to initiating therapy for the first time intrapartum [24]. The rapid HIV antibody test has been available in Canada since 2005 and is, in part, targeted towards use in this circumstance [25]. The rapid HIV antibody test is not widely available for use at most Canadian healthcare institutions; however, some jurisdictions are now investigating its use. Further efforts should be made towards implementing this test in the context of pregnant women presenting to labour and delivery with an unknown HIV status for the appropriate determination of HIV prophylaxis for their newborns.

Our study highlights associations with particular socioeconomic factors and ARV regimen in newborn HIV prophylaxis and therefore warrants further examination as to whether or not these factors unduly influence treatment decisions. Further analysis within this study already suggests that based on varying maternal presentations there is unequal distribution of socioeconomic factors and this may influence perinatal HIV transmission prophylaxis decisions. The results of this study can also contribute to research and practice pertaining to perinatally HIV-exposed infants by way of exploration of these influencing sociodemographic factors. With better assessment of perinatal HIV transmission risk factors, including use of rapid HIV antibody testing, the most optimal newborn ARV prophylaxis choice can be made, resulting in better care of these infants and appropriate use of multi-ARV regimens.

## Figures and Tables

**Table 1:** Summary of maternal demographics.

Maternal demographics	Proportion
Age (years)^*∗*^	31 (6)
Country of origin	
African	60 (49%)
Canadian	32 (26%)
Other	12 (10%)
Unknown	18 (15%)
HIV status known at delivery	
Y	93 (76%)
N	29 (24%)
Final HIV status	
Positive	86 (70%)
Negative	35 (29%)
Unknown	1 (1%)
Viral load of those who are HIV-positive (*n* = 86)	
<50 copies/mL	62 (72%)
50–1000 copies/mL	14 (16%)
>1000 copies/mL	5 (6%)
Unknown	5 (6%)
AZT intrapartum	87 (71%)
Type of delivery	
Vaginal	83 (68%)
Caesarean section	39 (32%)
Partner involved	
Y	67 (55%)
N	50 (41%)
Not documented	5 (4%)
HIV status of father of newborn	
Positive	26 (21%)
Negative	25 (20%)
Not documented	71 (58%)
Substance using	42 (34%)
Smoker	34 (28%)
Living situation	
Having their own home	97 (79%)
Homeless or living in shelter	25 (20%)
OHIP	97 (79%)
Social work referral	84 (69%)
Loss of custody of previous children	
Y	25 (20%)
N	51 (42%)
N/A	44 (36%)
Not documented	2 (2%)
Sex work	15 (12%)
Employed	
Y	33 (27%)
N	70 (57%)
Not documented	19 (16%)
Education (at least grade 12)	
Y	44 (36%)
N	13 (11%)
Not documented	65 (53%)

^*∗*^Continuous variable, reported in mean (SD).

**Table 2:** Summary of neonatal demographics.

Neonatal demographics	Proportion
Sex	
Male	63 (52%)
Triple therapy	37 (30%)
Gestational age (weeks)^*∗*^	38 (2)
Birth weight (g)^*∗*^	3013 (585)
Small for gestational age (<10th percentile)	21 (17%)
APGAR at 1 min <7	11 (9%)
APGAR at 5 min <7	3 (2%)
Toxicology screen	
Positive	33 (27%)
Negative	6 (5%)
N/A	83 (68%)
Of those that were toxicology screen positive (*n* = 33)	
Cocaine	31 (94%)
NICU admission	39 (32%)
Of those in NICU, preterm (<37 wks)	19 (49%)
Length of stay in hospital (days)	4.7 (7.6)
Meconium present at delivery	22 (18%)
CPS involved	41 (34%)
CPS apprehended	35 (29%)
Three most frequent cited reasons for triple therapy (*n* = 37)	
Maternal high risk behaviour	13 (35%)
Not documented	10 (27%)
High viral load	8 (22%)

^*∗*^Continuous variable, reported in mean (SD).

CPS: child protective services.

**Table 3:** Differences in maternal characteristics between newborn-monotherapy dyads and newborn-triple therapy dyads.

Maternal characteristic	Newborn-monotherapy dyads Mean (SE) or *n* (%)	Newborn-triple therapy dyads Mean (SE) or *n* (%)	*p* value
Age (years)^*∗*^	32 (0.7)	28 (1)	<0.01
Country of origin			
African	49 (66%)	11 (37%)	<0.01
Canadian	14 (19%)	18 (60%)	
Other	11 (15%)	1 (3%)	
Number of prenatal visits^*∗*^	8 (0.5)	4 (0.6)	<0.01
Final HIV status			
Positive	70 (83%)	16 (43%)	<0.01
Negative	14 (17%)	21 (57%)	
Viral load of those who are HIV-positive			
<50 copies/mL	59 (87%)	2 (15%)	<0.01
50–1000 copies/mL	9 (13%)	6 (46%)	
>1000 copies/mL	0 (0%)	5 (38%)	
Partner involved	50 (62%)	17 (47%)	NS
HIV status of father of newborn			
Positive	22 (59%)	4 (29%)	NS
Negative	15 (41%)	10 (71%)	
Substance use	17 (21%)	25 (69%)	<0.01
Smoker	14 (17%)	20 (67%)	<0.01
Living situation			
Own home	76 (89%)	21 (57%)	<0.01
Homeless or living in shelter	9 (11%)	16 (43%)	
Health insurance (OHIP)	68 (80%)	29 (78%)	NS
Social work referral	51 (60%)	33 (89%)	<0.01
Lost custody of previous children	8 (16%)	17 (68%)	<0.01
Sex work	3 (4%)	12 (34%)	<0.01
Employed	29 (41%)	4 (12%)	<0.01
Educated	36 (90%)	8 (47%)	<0.01
AZT intrapartum	70 (82%)	17 (47%)	<0.01

^*∗*^Continuous variable, reported in mean (SE).

**Table 4:** Differences in neonatal characteristics between newborn-monotherapy dyads and newborn-triple therapy dyads.

Neonatal characteristic	Newborn-monotherapy dyads Mean (SE) or *n* (%)	Newborn-triple therapy dyads Mean (SE) or *n* (%)	*p* value
Gender			
Male	45 (53%)	18 (49%)	NS
Gestational age (weeks)^*∗*^	38 (0.2)	37 (0.5)	<0.01
Birth weight (g)^*∗*^	3079 (63)	2864 (96)	NS
Small for gestational age	18 (21%)	3 (8%)	NS
Toxicology screen			
Positive	10 (12%)	23 (62%)	<0.05
NICU admission	19 (22%)	20 (54%)	<0.01
Length of stay in hospital (days)^*∗*^	3 (0.5)	8 (2)	<0.05
CPS involved	15 (18%)	26 (70%)	<0.01

^*∗*^Continuous variable, reported in mean (SE).

CPS: child protective services.

**Table 5:** Summary of groups in secondary analysis and differences in neonatal characteristics between groups.

Neonatal characteristic	Group 1 Mean (SD) or *n* (%)	Group 2 Mean (SD) or *n* (%)	Group 3 Mean (SD) or *n* (%)	Group 4 Mean (SD) or *n* (%)	*p* value
Distribution of groups	62 (52%)	22 (19%)	20 (17%)	14 (12%)	N/A
Newborn ARV regimen					
Monotherapy	60 (97%)	9 (41%)	9 (45%)	4 (29%)	<0.01
Triple therapy	2 (3%)	13 (59%)	11 (55%)	10 (71%)	
Gestational age (weeks)^*∗*^	39 (2)	37 (3)	38 (2)	37 (2)	<0.01
Birth weight (g)^*∗*^	3135 (581)	2985 (675)	2896 (390)	2669 (609)	<0.05
Small for gestational age	13 (21%)	1 (5%)	4 (20%)	3 (21%)	NS
Toxicology screen					
Positive	1^*∗∗*^	3 (75%)	15 (79%)	13 (93%)	NS
NICU admission	12 (19%)	9 (41%)	6 (30%)	12 (86%)	<0.01
Length of stay in hospital (days)^*∗*^	3 (3)	7 (14)	5 (5)	10 (9)	<0.01
CPS involved	1 (2%)	7 (32%)	18 (90%)	14 (100%)	<0.01

^*∗*^Continuous variable, reported in mean (SD).

^*∗∗*^Proportion not reported given low number of events.

CPS: child protective services.

**Table 6:** Differences in maternal characteristics between 4 groups in secondary analysis.

Maternal characteristic	Group 1 Mean (SD) or *n* (%)	Group 2 Mean (SD) or *n* (%)	Group 3 Mean (SD) or *n* (%)	Group 4 Mean (SD) or *n* (%)	*p* value
Age (years)^*∗*^	32 (6)	31 (6)	28 (6)	28 (8)	<0.01
Country of origin					
African	44 (77%)	15 (71%)	0 (0%)	0 (0%)	<0.01
Canada	6 (11%)	4 (19%)	11 (85%)	10 (100%)	
Other	7 (12%)	2 (10%)	2 (15%)	0 (0%)	
Number of prenatal visits^*∗*^	9 (3)	7 (4)	2 (3)	2 (2)	<0.01
Final HIV status					
Positive	62 (100%)	22 (100%)	1 (5%)	0 (0%)	<0.01
Negative	0 (0%)	0 (0%)	18 (95%)	14 (100%)	
Partner involved	39 (64%)	13 (65%)	8 (40%)	3 (25%)	<0.05
HIV status of father of newborn					
Positive	19 (56%)	4 (33%)	0 (0%)	0 (0%)	NS
Negative	15 (44%)	8 (67%)	1 (100%)	1 (100%)	
Substance use	4 (7%)	6 (29%)	17 (89%)	14 (100%)	<0.01
Smoker	2 (3%)	3 (17%)	15 (88%)	13 (100%)	<0.01
Living situation					
Own home	60 (97%)	20 (91%)	10 (50%)	3 (21%)	<0.01
Homeless or living in shelter	2 (3%)	2 (9%)	10 (50%)	11 (79%)	
Health insurance (OHIP)	49 (79%)	19 (86%)	13 (65%)	12 (86%)	NS
Social work referral	31 (50%)	16 (73%)	20 (100%)	14 (100%)	<0.01
Lost custody of previous children	1 (3%)	3 (21%)	11 (79%)	9 (82%)	<0.01
Sex work	0 (0%)	1 (5%)	6 (32%)	8 (57%)	<0.01
Employed	26 (47%)	4 (21%)	1 (6%)	0 (0%)	<0.01
Educated	27 (90%)	10 (100%)	4 (57%)	1 (14%)	<0.01
AZT intrapartum	60 (97%)	21 (95%)	4 (21%)	0 (0%)	<0.01

^*∗*^Continuous variable, reported in mean (SD).
